# Pediatricians’ oral health recommendations for 0- to 3-year-old children: results of a survey in Thuringia, Germany

**DOI:** 10.1186/1472-6831-14-44

**Published:** 2014-05-01

**Authors:** Yvonne Wagner, Roswitha Heinrich-Weltzien

**Affiliations:** 1 Department of Preventive Dentistry and Pediatric Dentistry, Jena University Hospital, Bachstr 18, Jena 07743, Germany

## Abstract

**Background:**

German societies of pediatricians and dentists disagree about oral health-related preventive recommendations (use of fluoride supplements, fluoride-containing toothpaste) for children aged 0–3 years. After failure to reach a consensus, there is no study that has evaluated the guidelines that pediatricians use in daily practice.

**Methods:**

A standardized questionnaire was sent to all 167 practicing pediatricians in the state of Thuringia, Germany, to assess the current oral health-related preventive recommendations. Data were analyzed using descriptive statistics.

**Results:**

The response rate was 52.0%. More than 9.0% of the pediatricians advise parents with regard to diet, use of baby bottles, oral hygiene and dental visits. The majority of pediatricians recommend to start tooth-brushing after the 1st birthday and recommend the use of toothpaste and a 1st dental visit after the 2nd birthday (78.0%). Additionally, 23.3% (n = 20) of pediatricians prescribe solely vitamin D, and 20.9% (n = 18) prescribe vitamin D combined with fluoride. Fluoride supplements are given as required by 37.2% (n = 32) of pediatricians, primarily between the 1st and 6th birthdays. The guidelines of the Pediatric Society were used by 1.2% of the pediatricians, the guidelines of the dentists were used by 5.8%, and a mix of both was used by 93.0%. The simultaneous use of fluoride supplements and fluoride toothpaste in the first three years was recommended by 45.9% of the pediatricians.

**Conclusions:**

Pediatricians’ oral health recommendations are based on a mix of the guidelines from the German societies of pediatricians and dentists and led to no use or possible overdose of fluoride. Against the background of early childhood caries and dental fluorosis, there is a need for uniform guidelines.

## Background

Since the publication of the national guideline *Fluoridation –recommendations for caries prevention* by the German Society of Oral and Maxillofacial Surgery (DGZMK) in 2005, there have been numerous discussions between pediatricians and dentists regarding the use of fluoride tablets and fluoride-containing toothpaste [1].

The German Society of Pediatrics and Adolescent Medicine (DGKJ) and the German Academy for Pediatrics and Adolescent Medicine (DAKJ) reject the use of toothpaste, particularly fluoride-containing toothpaste, for infants and toddlers up to the age of 4 years and recommend the use of fluoride supplements as drops or tablets [2].

The DGZMK agrees with other societies such as the European Academy of Pediatric Dentistry (EAPD) and the American Academy of Pediatric Dentistry (AAPD) and prefers the early use of fluoridated toothpaste for tooth-brushing [1,3,4].

This disagreement over the preventive regime in the first 3 years of life leads to confusion in parents, primary health care providers, pediatricians, family physicians and dentists about the benefits of fluoride. Even in 2013, no agreement could be reached between the German societies regarding the different viewpoints about the preference for topical fluoride application versus systemic fluoridation by tablets [5].

Dental caries as a non-communicable disease is acknowledged as major global public health issue. Early childhood caries (ECC) is the most common chronic disease in children [6,7]. The use of fluoride for caries prevention has been evidence-based for decades and is the main reason for the decline in caries [1-5].

Despite several initiatives to improve oral health in childhood, the reduction of caries prevalence in the primary dentition has been quite modest [7]. A recent representative study in Germany showed that there was no significant reduction of caries prevalence in 6- and 7-year-old children in last 20 years [8]. Actually, the federal state of Thuringia is in the bottom position regarding oral health in children [8].

Worldwide, there is a demand for establishment of dental care by 12 months of age [9]. In Germany, the first dental visit and examination are recommended by health insurance providers to take place between 30 and 42 months of age. Unlike dentists, pediatricians see the majority of children periodically during their first years of life and have the opportunity to sensitize parents to the oral health of their children. Primary care providers play an important role in healthy growth and can provide anticipatory guidance and preventive counseling about oral hygiene, diet and fluoride exposure [10,11].

Considering this background, we wanted to determine which current preventive recommendations related to oral health in children aged 0 to 3 years are implemented in daily practice by pediatricians in Thuringia and which guidelines they follow. As a secondary objective of this study, we wanted to examine whether there are differences between pediatricians in terms of oral health practices after taking their characteristics (gender, age, professional experience, employee or practice owner, rural or urban practice) into account. Finally, the age at which pediatricians in Thuringia refer patients to a dentist was determined.

## Methods

### Study population

A survey among pediatricians in Thuringia was conducted using a standardized questionnaire that could be returned by mail, fax or email. The study population was identified through the database of the Thuringia Association of Health Insurance Doctors and the public phonebook. The eligibility criteria were non-retired pediatricians currently practicing in Thuringia. A questionnaire with a cover letter, postage-paid envelope and information about the possibility of using fax or email was mailed to each of the 167 pediatricians practicing in the state of Thuringia in February 2009, with data collection beginning immediately thereafter. Non-respondents to the original mailing received a reminder by post and fax. No incentives were offered. The survey was closed on April 30, 2009.

The completed questionnaires were entered in excel files by two separate individuals and cross-compared.

The Ethics Committee of Jena University Hospital approved this study (3688-02/13).

### Questionnaire

The survey instrument was a 33-item standardized questionnaire that took approximately 10 minutes to complete. The questionnaire included closed questions with predetermined answers and open-ended questions. The open questions allowed a differentiated analysis. The first portion included 14 questions about prescription of vitamin supplements, defining the beginning and end of consumption and the method of administration and dosage.

The second portion contained 8 questions about recommended oral hygiene measures, and the third included 6 questions about the counseling of parents and referrals to the dentist. The questionnaire also collected information about the pediatricians’ characteristics with regard to gender, age, work experience, whether the pediatrician was an employee or practice owner as well as whether the practice was rural or urban. The questionnaire was tested in a review group of 10 randomly selected pediatricians in Thuringia regarding comprehensibility, mistakes and time to completion before being sent. The revised questionnaire was then posted to the other 157 pediatricians in Thuringia.

### Data analysis

Data were analyzed using descriptive statistics. Pediatricians’ responses were compared using the Freeman Halton test. The Freeman Halton test is an extension of Fisher’s exact test to compute dichotomous samples. Differences were considered statistically significant for *p* values < 0.05.

## Results

**Figure 1:**
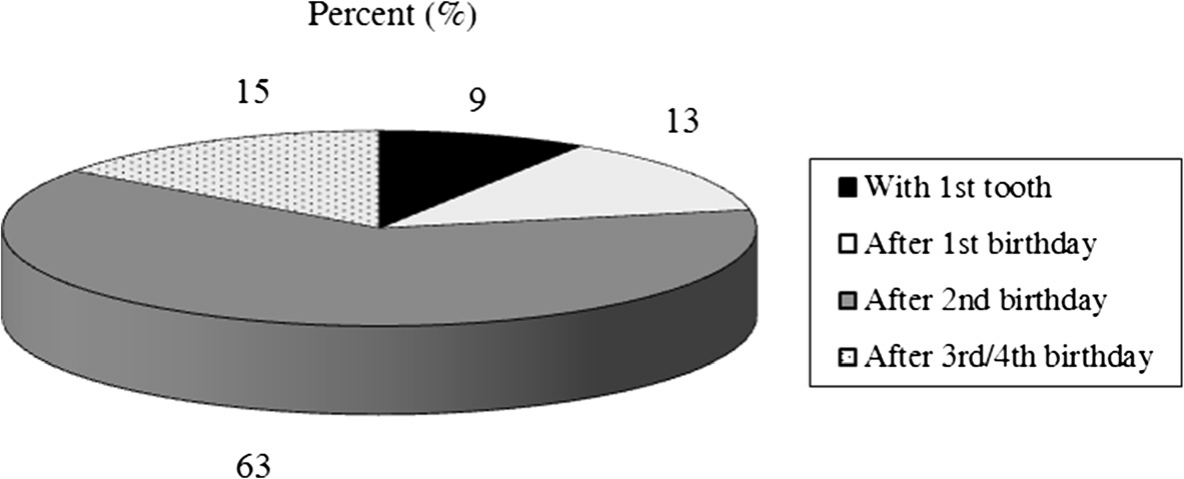
Pediatricians’ recommendations for the 1st dental visit.

**Figure 2:**
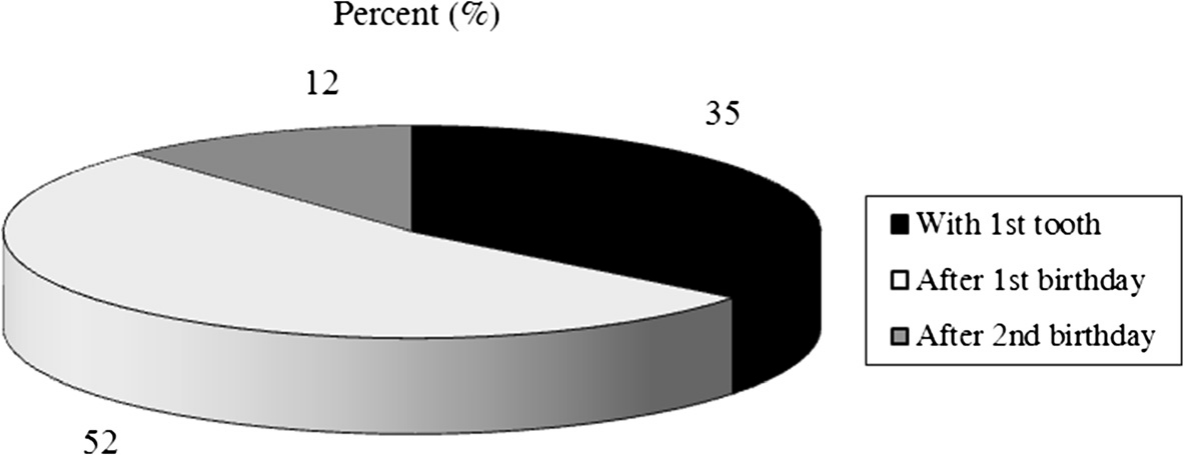
Pediatricians’ recommendations for the start of tooth-brushing.

**Figure 3:**
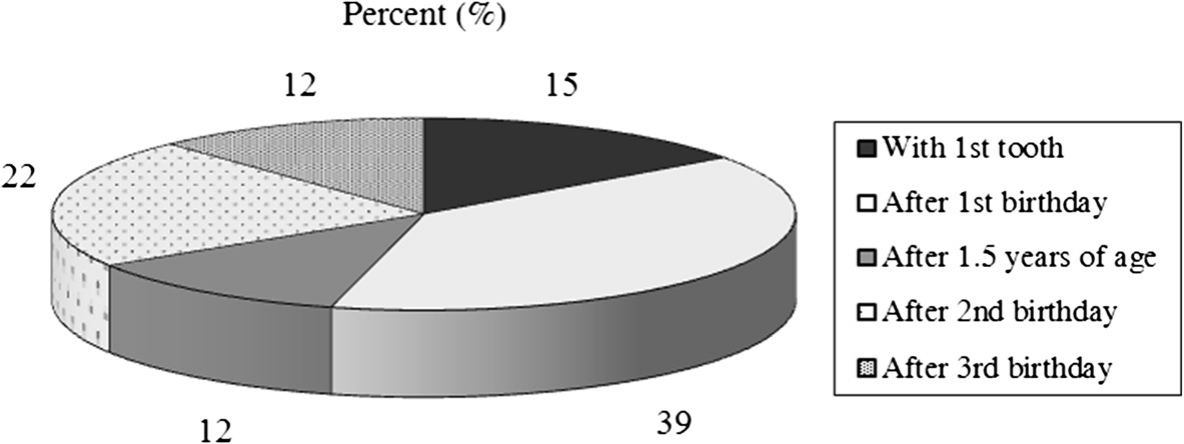
Pediatricians’ recommendations for the start of the use of toothpaste.

The response rate was 52.0% and was not significantly different between males and females. A total of 76.7% of the responders were female, and 23.3% were male with a mean age of 43.5 ± 9.2 years; 64.0% of the pediatricians were > 40 years old and had more than 10 years of professional experience. It was found that 27.9% were employees, and 72.1% were self-employed. The majority practiced in an urban region (67.4%). Nearly all pediatricians (90.0%) advised parents about diet, use of baby bottles, oral hygiene and dental visits. Most pediatricians (81.2%) recommended an age for the first dental visit (Figure 1), and 66.3% referred depending on dental findings. The majority of pediatricians recommended the start of tooth-brushing and the use of toothpaste after the 1st birthday (Figures 2, 3). Furthermore, 9.0% offered advice about the frequency of tooth brushing, 20.9% about the dosage of toothpaste, and 93.0% recommended fluoride toothpaste. Almost one quarter (23.3%) of the pediatricians prescribed solely vitamin D, and 20.9% prescribed vitamin D combined with fluoride immediately after birth. Fluoride tablets were given as required by 37.2% of pediatricians, primarily between the 1st and 6th birthdays (Table 1). Few (1.2%) pediatricians used the guideline of the Pediatric Society, 5.8% used the guideline of the dentists, and 93.0% used a mix of both. Finally, 45.9% of the pediatricians recommended the simultaneous use of fluoride supplements and fluoride toothpaste in the first three years without information about other fluoride sources or water fluoride level.

**Table 1:** Pediatricians’ preventive recommendations regarding vitamin supplements

**Prescription of vitamin supplements**			**Pediatricians (%)**
**Vitamin D**	**Vitamin D combined with fluoride**	**Fluoride tablets**	
Yes	No	No	**23.5**
No	Yes	No	**21.2**
Yes	Yes	Yes	**21.1**
Yes	Yes	No	**18.9**
No	Yes	Yes	**14.1**
Yes	No	Yes	**1.2**

The preventive recommendations were given regardless of the gender and age of the pediatrician (p > 0.05) and tended to differ between pediatricians practicing in rural or urban areas regarding the recommendation of toothpaste (p < 0.001) and the use of vitamin supplements (p < 0.05). The answers of employees and practice owners were significantly different regarding the recommendations for the timing of commencement of tooth-brushing (p < 0.001), the use of toothpaste (p < 0.001), the use of vitamin supplements (p < 0.001) and the recommended age for the first dental visit (p < 0.05) (see Additional file 1 and Additional file 2).

## Discussion

The results of our study confirmed the disagreement regarding the oral health-related preventive recommendations among pediatricians. There were significant differences between the recommended age for the start of tooth-brushing, the use of toothpaste and prescription of vitamin D with or without fluoride. These results refer to the professional recommendations. Additionally, the study findings showed a large variability regarding the recommended age for the start of tooth-brushing and the use of toothpaste. The majority of pediatricians did not use the specified recommendations "with the 1st tooth" or "after 4th birthday" of the professional associations. The guidelines of the DGKJ and the DAKJ suggest the prescription of fluoride supplements immediately after birth and starting the use of toothpaste by 4 years of age if the child can spit it out [2,5]. The DGZMK prefers the start of tooth-brushing with fluoride toothpaste when the first tooth erupts instead of using fluoride supplements [1,5]. Between these guidelines are 3 years of disagreement, and only a small group of pediatricians adhere to them. The majority of pediatricians who responded to this questionnaire used a mix of both current guidelines with a result of no use or possible overdose of fluoride in children.

A comparison of our results with nationally and internationally conducted surveys among pediatricians showed that there were commonalities as well as differences [12-18].

One commonality was the recommended age for the first dental visit. Most pediatricians refer children to the dentist at the age of 3 [12-18]. The majority of respondents from our study recommended the first dental visit after the 2nd (63.0%) or 3rd and 4th (15.0%) birthday. The National German Health Interview and Examination Survey for Children and Adolescents showed that 92.3% of children had their first dental visit within the first 3 years of life and confirm our results [19]. Internationally, there is a demand for early establishment of regular dental care by age one [9]. In most cases, parents do not take the initiative to make sure that their children receive regular dental care in first years of life. Irregular dental visits are common, and toothache is the major driving factor for seeking dental care [9]. Until this process is reevaluated in the minds of parents, pediatricians and dentists, the pediatrician will regard her/himself as being responsible for oral health care in a child’s first years of life.

In other countries, there is agreement between the societies of pediatricians and dentists that tooth-brushing with fluoride toothpaste has to begin with the eruption of the first tooth [12,13,20,21]. A survey among pediatricians in Connecticut showed that most of them recommended this starting point [13]. In contrast, the majority of our responders recommended the start of tooth-brushing and the use of toothpaste after the 1st birthday. Pediatricians and dentists are in agreement that young children swallow the majority of toothpaste until they can spit out and that it is necessary to limit the consumption of toothpaste with dose recommendations [1-5,13,20-22]. Additionally, parents should assist and supervise tooth-brushing [3-5,21,22]. The AAPD recommends that children less than 2 years of age should use a smear layer of fluoride toothpaste (≥1000 ppm) twice a day, and children up to 5 years of age should use a pea-sized amount [4]. The EAPD recommendations suggest that children use a pea-sized amount of fluoride toothpaste twice daily, with the difference that children less than 2 years of age should brush with 500 ppm fluoride toothpaste, and children up to 6 years of age should brush with 1000 ppm fluoride toothpaste due to the risk of dental fluorosis (DF) [3]. In Germany, the regulations go a step further, suggesting that children up to the age of 6 should use 500 ppm fluoride toothpaste to minimize the cumulative effects of other fluoride sources [1,5].

In contrast, only 20.9% of our responders advised parents regarding the dosage of fluoride toothpaste and 9.0% regarding the frequency of tooth-brushing. Additionally, 45.9% of pediatricians recommended the simultaneous use of fluoride supplements and fluoride toothpaste in first three years without information about other fluoride sources or water fluoride levels. A questionnaire among German dentists showed similar results [23]. Most of the dentists (92.0%) recommended tooth-brushing with fluoride toothpaste, but one fifth of them also prescribed fluoride supplements, and 58% of the respondents did not know the dose limit of 0.05 milligrams of fluoride per kilogram body weight for the development of DF [23].

Children younger than 3 years are considered to be at risk for DF of permanent incisors and first molars [1,2,5,21,22,24]. In Germany, the prevalence of DF (mild form) in 7-year-old children is approximately 10.1% [24]. The increase in the number of sources of fluoride exposure, such as water, beverages, food, salt, fluoride supplements and dental products, could lead to an overdose in this sensitive phase of tooth development [1,2,5,21,22,24]. Reviews had found that prolonged use of fluoride supplements during tooth development increases the risk of developing DF [22,25]. If tooth-brushing with fluoride toothpaste started later than 12 months of age or the fluoride level of toothpaste was < 1000 ppm, a statistically significant reduction in DF was found [22].

Both guideline recommendations of the DAKJ and the DGZMK cannot exclude the risk of developing DF. Studies about the effectiveness in the primary dentition of fluoride supplements and fluoride toothpaste containing <1000 ppm are weak and are difficult to perform for ethical reasons, so discussions between both societies will be continued [1-5,21,22,25].

Healthcare professionals want to promote healthy development of children, and guidelines should encourage them to treat and inform patients to the best of the current knowledge and belief. For the prevention of ECC as a public health problem, adverse effects of fluoride use such as DF with purely aesthetic problems should remain in the background. So that an agreement can be reached between pediatricians and dentists, all healthcare professionals should respect the opinions of other professionals and pay attention to daily fluoride ingestion, especially in the first 3 years, and use either fluoride tablets or fluoride toothpaste for children with no increased risk of caries [1-5,21,22,24].

As a secondary objective of this study, we wanted to examine whether there were differences between pediatricians in terms of oral health practices when taking their characteristics into account. This study found that the preventive recommendations given were irrespective of the gender and age of the pediatrician but tended to differ between pediatricians practicing in rural and urban areas. Pediatricians practicing in urban areas were more progressive and recommended vitamin D without fluoride and the use of toothpaste much earlier than their colleagues from rural areas. The preventive recommendations of employees and practice owners were significantly different. The majority of practice owners recommended commencement of tooth-brushing with the first tooth and the use of fluoride supplements. The recommendations to use toothpaste as well as the first dental visit were made in later years. This reflects recommendations that have been made for decades [2]. Employees’ university studies were usually relatively recent, and their recommendations were likely based on recent international publications and guidelines.

Actually, oral health plays a minor role in the medical curriculum, and several studies have revealed that pediatricians’ knowledge regarding child oral health is insufficient [12-18].

To win the battle against ECC and to improve the oral health of our children, pediatricians as well as dentists are challenged to incorporate infant oral health care into their practices [6,7,9-11,20,25,26]. Training programs should encourage these healthcare professionals to assess caries risk, to perform a knee-to-knee exam and to counsel parents about oral hygiene, fluoride, diet (especially with regard to sugared and acidic drinks) and prevention of early transference of bacteria from parents to child [10,11,20,25,26].

Until now, there have been no special oral health training programs for pediatricians in Germany. Internationally, such training programs already exist [27,28]. A study showed that after the implementation of a caries prevention program in the medical education curriculum, physicians were able to diagnose dental caries, and it became the 11th most common diagnosis made in the clinic [28]. Infant oral health training courses for primary care providers could be the basis for successful collaborations among pediatricians and dentists.

There are a few limitations of this study. This was a regional survey conducted in one of the federal states of Germany, Thuringia, so the results are not representative of the rest of Germany. For the evaluation and generalizability of our results, we compared them with national and international studies of pediatricians that achieved similar results and confirmed our study findings [12-21].

Another limitation of this study was the response rate. On the one hand, the content of a study called "Use of fluorides and oral health related recommendations in 0- to 3-year-old children" is a tiresome problem due to years of dispute, and on the other hand, it is an internationally increasing problem to achieve high return rates [29-35]. The Commonwealth Fund conducted an international survey of doctors from 7 countries and achieved a participation rate in Germany of 18.1% in 2006 and 31% in 2008 [29,30]. To improve the response rate of our survey, pediatricians had the ability to return their responses by fax, email or in postage-paid envelopes, and non-respondents received a reminder. In this way, we were able to achieve a response rate of 52.0%. Typical return rates for postal surveys in Germany are between 10 and 60 percent [31]. Higher rates could be achieved with more effort and higher costs, but the cost-benefit ratio was not justified with this study. Of course, a response rate of 52.0% means that approximately 48.0% of the practitioners did not respond and that there could be potential bias. A review of the literature showed that low response rates to physician surveys are common and do not imply non-response bias [32]. A survey in the USA showed that physicians have more homogeneous characteristics than other professional groups and that non-response bias is likely to be less important [33]. A meta-analysis of the impact of non-response rates on non-response bias of 566 standard estimates from 44 studies showed that non-response rates could explain 8 to 19 percent of the variation in different estimates of the non-response bias [34]. Another study examined a set of 30 studies estimating the non-response bias of descriptive statistics and found that the non-response rate is a poor predictor of bias magnitude [35]. In this study, no analysis of the group of non-responders was made due to cost and effort. A comparison with other studies of pediatricians and a review of the literature about non-response rates and non-response bias has shown that no other results could be reasonably expected [12-18,29-35].

To our knowledge, this was the first survey examining the guidelines used by Thuringian pediatricians in daily practice. The results revealed that uniform guidelines have to be developed containing exact information and instructions about when and what should be done, prescribed or recommended, and how it should be done. DF is an increasing aesthetic problem, but the consequences of ECC affect general and oral health as well as quality of life in children [7,9].

In summary, it can be stated that all healthcare professionals should be trained in infant oral health care to fulfill the requirement of the Global Caries Initiative of the FDI World Dental Federation, which places primary oral health care within maternal and child health programs using multi-sectoral and collaborative approaches [6].

## Conclusions

Thuringian pediatricians’ oral health-related preventive recommendations are a mix of current guidelines from both professional societies and lead to no use or possible overdose of fluoride in children. Against the background of ECC and DF, there is a need for a uniform guideline in Germany.

## Competing interests

The authors declare that they have no competing interests.

Financial competing interests

The authors declare that they have no financial competing interests.

Non-financial competing interests

The authors declare that they have no non-financial competing interests.

## Authors’ contributions

YW and RHW are responsible for the reported research. Both authors have made substantial contributions to study conception and design, analysis and interpretation of data and drafting and revising the paper critically for important intellectual content. Both authors read and approved the final manuscript.

## Pre-publication history

The pre-publication history for this paper can be accessed here:

http://www.biomedcentral.com/1472-6831/14/44/prepub

## Supplementary Material

Additional file 1 Pediatricians’ preventive recommendations depending on gender and age (proportions in percent). Click here for file

Additional file 2 Pediatricians’ preventive recommendations depending on employment relationship and location of the practice (proportions in percent). Click here for file
