# Supplementary material for: Pediatricians’ oral health recommendations for 0- to 3-year-old children: results of a survey in Thuringia, Germany
Source: BMC Oral Health. 2014 May 1;14:44. doi: 10.1186/1472-6831-14-44 (PMC4021417; doi:10.1186/1472-6831-14-44)
Supplement: Additional file 1 — Pediatricians’ preventive recommendations depending on gender and age (proportions in percent). [file 1472-6831-14-44-S1.doc]

**Additional file 1:** Pediatricians` preventive recommendations depending on gender and age (proportions in percent)

| **Recommendations** | | **Gender** | | **p-Value** | **Age** | | **p-Value** |
| --- | --- | --- | --- | --- | --- | --- | --- |
|  |  | **Male**  **(n=20)** | **Female (n=66)** |  | **< 40 years**  **(n=31)** | **> 40 years**  **(n=55)** |  |
| **Tooth-brushing** | **With 1st tooth** | 25.0 | 37.9 | 0.543 | 35.5 | 34.5 | 0.949 |
|  | **After 1st birthday** | 60.0 | 48.5 |  | 48.4 | 52.7 |  |
|  | **Later** | 15.0 | 10.6 |  | 12.9 | 10.9 |  |
|  | **No age specification** | 0.0 | 3.0 |  | 3.2 | 1.8 |  |
| **Toothpaste** | **With 1st tooth** | 10.0 | 15.2 | 0.882 | 16.1 | 12.7 | 0.631 |
|  | **After 1st birthday** | 40.0 | 37.9 |  | 38.7 | 38.2 |  |
|  | **Later** | 45.0 | 43.9 |  | 35.5 | 49.1 |  |
|  | **No age specification** | 5.0 | 3.0 |  | 9.7 | 0.0 |  |
| **Supplements** | **Solely Vitamine D** | 15.0 | 25.8 | 0.452 | 29.0 | 20.0 | 0.304 |
|  | **Solely Vitamine D combined with fluoride** | 30.0 | 18.2 |  | 25.8 | 18.2 |  |
|  | **Individual** | 55 | 56 |  | 45.2 | 61.8 |  |
|  | **Fluoride tablets as required** | 40.0 | 36.4 |  | 35.5 | 38.2 |  |
| **1st dental visit** | **With 1st tooth** | 5.0 | 7.6 | >0.999 | 3.2 | 9.2 | 0.721 |
|  | **After 1st birthday** | 5.0 | 12.1 |  | 6.4 | 12.7 |  |
|  | **Later** | 50.0 | 65.1 |  | 58.1 | 63.6 |  |
|  | **No age specification** | 40.0 | 15.2 |  | 32.3 | 14.5 |  |
|  | **Dependent on dental findings** | 70.0 | 62.1 |  | 74.2 | 58.2 |  |
