# Supplementary material for: Pediatricians’ oral health recommendations for 0- to 3-year-old children: results of a survey in Thuringia, Germany
Source: BMC Oral Health. 2014 May 1;14:44. doi: 10.1186/1472-6831-14-44 (PMC4021417; doi:10.1186/1472-6831-14-44)
Supplement: Additional file 2 — Pediatricians’ preventive recommendations depending on employment relationship and location of the practice (proportions in percent). [file 1472-6831-14-44-S2.doc]

**Additional file 2:** Pediatricians` preventive recommendations depending on employment relationship and location of the practice (proportions in percent)

| **Recommendations** | | **Employment relationship** | | **p-Value** | **Practice location** | | **p-Value** |
| --- | --- | --- | --- | --- | --- | --- | --- |
|  |  | **Employee**  **(n=24)** | **Owner**  **(n=62)** |  | **Rural area (n=28)** | **Urban area (n=58)** |  |
| **Tooth-brushing** | **With 1st tooth** | 12.5 | 43.5 | **0.000** | 32.1 | 36.2 | 0.159 |
|  | **After 1st birthday** | 87.5 | 37.2 |  | 42.9 | 55.2 |  |
|  | **Later** | 0.0 | 16.1 |  | 21.4 | 6.9 |  |
|  | **No age specification** | 0.0 | 3.2 |  | 3.6 | 1.7 |  |
| **Toothpaste** | **With 1st tooth** | 8.3 | 16.1 | **0.000** | 0.0 | 20.7 | **0.000** |
|  | **After 1st birthday** | 87.5 | 19.4 |  | 14.3 | 50.0 |  |
|  | **Later** | 4.2 | 59.7 |  | 78.6 | 27.6 |  |
|  | **No age specification** | 0.0 | 4.8 |  | 7.1 | 1.7 |  |
| **Supplements** | **Solely Vitamine D** | 58.3 | 9.7 | **0.000** | 3.6 | 32.8 | **0.005** |
|  | **Solely Vitamine D combined with fluoride** | 37.5 | 14.5 |  | 21.4 | 20.7 |  |
|  | **Individual** | 4.2 | 75.8 |  | 75.0 | 46.5 |  |
|  | **Fluoride tablets as required** | 0.0 | 51.6 |  | 53.6 | 29.3 |  |
| **1st dental visit** | **With 1st tooth** | 0.0 | 9.7 | **0.048** | 0.0 | 10.3 | 0.252 |
|  | **After 1st birthday** | 4.2 | 12.9 |  | 7.1 | 12.2 |  |
|  | **Later** | 87.5 | 51.6 |  | 64.3 | 60.3 |  |
|  | **No age specification** | 8.3 | 25.8 |  | 28.6 | 17.2 |  |
|  | **Dependent on dental findings** | 75.0 | 59.7 |  | 53.6 | 69.0 |  |
